# *Artemisia annua* L. Extracts Irreversibly Inhibit the Activity of CYP2B6 and CYP3A4 Enzymes

**DOI:** 10.3390/biomedicines11010232

**Published:** 2023-01-16

**Authors:** Martin Kondža, Marta Mandić, Ivona Ivančić, Sanda Vladimir-Knežević, Ivica Brizić

**Affiliations:** 1 Faculty of Pharmacy, University of Mostar, Matice Hrvatske bb, 88000 Mostar, Bosnia and Herzegovina; 2 Department of Pharmacognosy, Faculty of Pharmacy and Biochemistry, University of Zagreb, Trg Marka Marulića 20, 10000 Zagreb, Croatia; 3 University Clinical Hospital Mostar, Kralja Tvrtka bb, 88000 Mostar, Bosnia and Herzegovina

**Keywords:** *Artemisia annua* L., CYP2B6, CYP3A4, methanolic extracts, inhibition, heme

## Abstract

*Artemisia annua* L. has long been known for its medicinal properties and isolation of ingredients whose derivatives are used for therapeutic purposes. The CYP2B6 and CYP3A4 enzymes belong to a large family of cytochrome P450 enzymes. These enzymes are involved in the metabolism of drugs and other xeonobiotics. It is known that various compounds can induce or inhibit the activity of these enzymes. The aim of this study was to investigate the nature of the inhibitory effect of *Artemisia annua* extract on CYP2B6 and CYP3A4 enzymes, as well as the type of inhibition, the presence of reversible or pseudo-irreversible inhibition, and the possible heme destruction. The methanolic extract of *Artemisia annua* showed an inhibitory effect on CYP2B6 (by almost 90%) and CYP3A4 enzymes (by almost 70%). A significant decrease in heme concentration by 46.8% and 38.2% was observed in different assays. These results clearly indicate that the studied plant extracts significantly inhibited the activity of CYP2B6 and CYP3A4 enzymes. Moreover, they showed irreversible inhibition, which is even more important for possible interactions with drugs and dietary supplements.

## 1. Introduction

**Figure 1:**
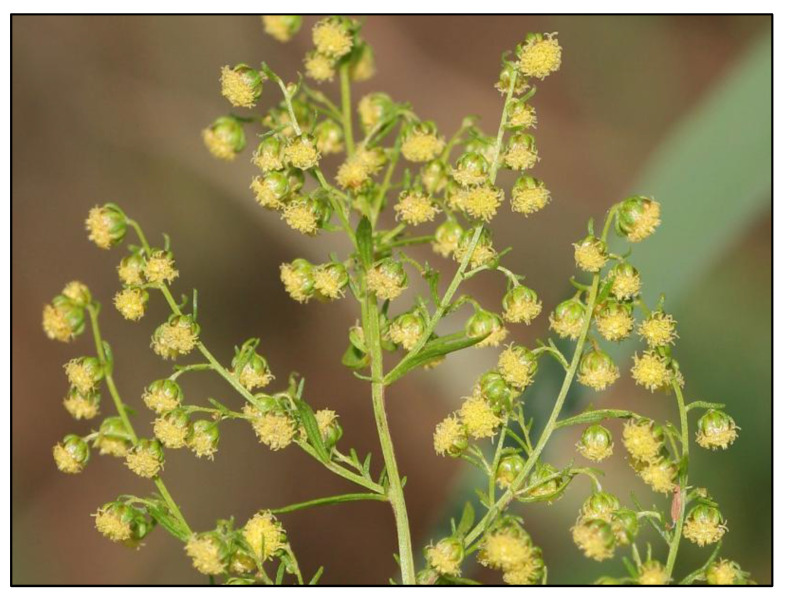
*Artemisia annua* L. [4].

Although 50 years have passed since the discovery of artemisinin [1], the pharmacologically and biologically active compounds of the *Artemisia annua* L. (sweet wormwood) continue to engage and fascinate scientists around the world. *Artemisia annua* (Figure 1) belongs to the annual herbaceous herb class, and grows in Asia and India as well as some parts of Europe, America, Africa, and Australia [2,3].

Sweet wormwood is a plant that people use on many occasions, from nutrition to health purposes. In parts of Asia, this plant is used as a spice, tea, or pressed juice [5]. It has been used as a medicine in traditional Chinese medicine for centuries. In addition, the official Chinese pharmacopoeia describes an infusion of the dried parts of the plant as a remedy for malaria and fever [6]. In addition to its antimalarial activity, there are numerous studies showing the antimicrobial, anticholesterol, antiviral, anti-inflammatory, anti-plasmodial, antitumor, antiobesity, and anticonvulsant effects of this plant [6,7,8,9,10,11,12]. The reason why sweet wormwood has such a wide range of effects on different types of diseases is that it contains numerous biologically active groups of compounds in its composition (Table 1).

In 2015, the Chinese pharmaceutical scientist Tu Youyou was awarded the Nobel Prize in Physiology or Medicine for the discovery and isolation of artemisinin, which is believed to be responsible for the antimalarial activity of this plant. According to its chemical structure, artemisinin belongs to the sesquiterpenes, a class of terpenes with three isoprene units. In addition to artemisinin, sweet wormwood contains a handful of other constituents that exhibit a range of biological and pharmacological effects [13].

Cytochrome P450 (CYP) enzymes are a large family of enzymes found in all biological kingdoms, including humans. In humans, the CYP enzymes are located in the mitochondria or endoplasmic reticulum. This large group of enzymes are monooxygenases; they require an external oxygen donor to function properly. According to their structure, CYP enzymes are hemoproteins; they have heme as a prosthetic group [14]. CYP enzymes are involved in the metabolism of xenobiotics, including drugs that humans ingest daily. When metabolized by the same CYP enzymes, numerous substances can act as inducers or inhibitors of enzymatic activity [15].

The CYP2B6 enzyme is expressed in the liver and to some extent in the lungs [16]. It is an enzyme involved in approximately 5% of reduction and oxidation reactions within the CYP group [17]. The marker reaction used to measure the activity of this enzyme is the *N*-demethylation of *S*-mephenytoin. Efavirenz, which converts CYP2B6 to hydroxylated metabolites, is also used for this purpose [18]. Other substrates of this enzyme include artemisinin, bupropion, cyclophosphamide, ifosfamide, and methadone. The following compounds have been shown to inhibit of this enzyme: 17α-ethinylestradiol, duloxetine, thiotepa, ticlodipine, clopidogrel, methadone, sibutramine, and ritonavir [18,19]. Besides drugs, numerous compounds of natural origin also inhibit the activity of this enzyme very successfully [18,20].

CYP3A4 is the most important drug-metabolizing enzyme in the CYP group, as it metabolizes about 33% of drugs. It is an enzyme responsible for 20% of all reduction and oxidation reactions within the CYP group [17]. Since it has a large active site, CYP3A4 is involved in the metabolism of numerous xenobiotics. The reaction marker used to measure the activity of this enzyme is the hydroxylation of testosterone to 6β-hydroxytestosterone [18]. Some of the substrates for this enzyme are clarithromycin, erythromycin, cisapride, astemizole, verapamil, atorvastatin, lovastatin, simvastatin, and sildenafil. Ritonavir, diltiazem, cimetidine, amiodarone, verapamil, and many other drugs act as inhibitors of this enzyme [18]. It has also been found that numerous substances of natural origin, such as flavonoids, can also significantly inhibit the activity of the CYP3A4 enzyme [20,21] and lead to clinically significant and potentially dangerous interactions [22].

The aim of this study was to investigate the inhibitory effect of *Artemisia annua* L. extract (AAE) on the activity of CYP2B6 and CYP3A4 enzymes, as well as to examine heme binding and pseudo-irreversible and reversible inhibition.

## 2. Materials and Methods

### 2.1. Materials

The aerial parts of the *Artemisia annua* L. plant were purchased on the market (Suban, Croatia). Cytochromes P450 2B6 and 3A4 (recombinant) were co-expressed with nicotinamide-adenine-dinucleotide phosphate (NADPH) reductase and obtained from Thermo Fisher Scientific (Waltham, MA, USA), as well as cytochrome b5 in baculosomes. The contents of CYP2B6 and CYP3A4 were confirmed to be 1 μM, as was declared by the manufacturer, based on the cytochrome P450 carbon monoxide assay [23]. β-nicotinamide-dinucleotide phosphate disodium salt (NADP^+^), glucose-6-phosphate (G6P), and glucose-6-phosphate dehydrogenase (G6PDH) obtained from Sigma-Aldrich (St. Louis, MO, USA) were used to prepare the generation system. Formic acid (85%, p.a.) was purchased from Semikem (Sarajevo, Bosnia and Herzegovina), potassium phosphate (p.a.) and dichloromethane (p.a.) from Kemika (Zagreb, Croatia), methanol from Merck KGaA (Darmstadt, Germany), ultrapure water from Sigma-Aldrich, and acetonitrile from KEFO (East Sarajevo, Bosnia and Herzegovina). In order to prepare a potassium phosphate buffer of pH 7.4, a potassium dihydrogen phosphate obtained from Kemika d.d. was used. Sodium hydroxide (Semikem d.o.o.) was used to adjust the pH. For the marker reactions for CYP2B6 and CYP3A4, *S*-mephenytoin, *S*-nirvanol, testosterone, and 6β-hydroxytestosterone from Sigma Aldrich were used. Clopidogrel and troleandomycin, used as positive controls for enzyme activities, were obtained from the Agency for Medicines and Medical Devices of Bosnia and Herzegovina (Banja Luka, Bosnia and Herzegovina). Pyridine (p.a.) (Semikem d.o.o.), bovine hemin (Sigma-Aldrich), and dimethylsulfoxide (DMSO) (Semikem d.o.o.) were used in the hemochromopyridine assay. Potassium hexacyanoferrate (PCF) (Siegfried AG, Zofingen, Switzerland) and diltiazem (Enzo Life Sciences, Farmingdale, NY, USA) were used in the study of reversible and pseudo-reversible inhibition. Plant extracts were shaken on an orbital shaker with mat platform (Sigma-Aldrich). Superoxide dismutase (SOD) (Sigma-Aldrich), catalase (CAT) (Sigma-Aldrich), and hydrogen peroxide (36%, p.a.) (Semikem) were used to test out the binding specificity of the enzyme. A water bath (Thermo Fisher Scientific) was used for enzyme incubations. Samples were centrifuged using the FC5306 mini centrifuge (OHAUS, Parsippany, NJ, USA). Assay of residual enzyme activity was performed using high-performance liquid chromatography coupled with UV-Vis detection (HPLC UV-Vis, Agilent 1100, Agilent Technologies, Santa Clara, CA, USA). To record the sample spectra, a spectrophotometer UV-1280 (Shimadzu Corporation, Kyoto, Japan) was used. In order to retrieve the enzyme activity, Slide-A-Lyzer Dialysis Cassettes (Thermo Fisher Scientific) were used.

### 2.2. Plant Extraction

The extract of *Artemisia annua* (AAE) was obtained according to the method described by Mashati et al. [24]. The aerial parts of the plant were macerated with methanol. Then, the plant parts were left in the methanol overnight at room temperature in an orbital shaker. The macerate was then filtered, and the filtrate was evaporated to dryness. Next, 100 mg of the extract was dissolved in 1 mL of DMSO, and then the desired concentrations of the extract were prepared by dissolving in methanol.

### 2.3. Determination of Residual Activity

In order to determine the nature of the inhibitory effect of AAE on CYP2B6 and CYP3A4, three types of inhibition assays were performed: direct, time-dependent, and metabolic inhibition assays of the enzyme. In the direct inhibition assay, no preincubation of AAE with the enzyme was performed. The generating system was added directly to the incubation mixture with the substrate and incubated for 15 min. In the time-dependent inhibition assay, AAE was first pre-incubated with the enzyme in a water bath for 30 min, and then the reaction was started by adding the generating system and substrate. The samples were then incubated for an additional 15 min. In the metabolism-dependent inhibition assay, AAE, enzymes, and the generating system were first pre-incubated for 30 min, and the reaction started by adding the substrate. The samples were incubated for an additional 15 min. Enzyme incubations were performed in triplicate, with mechanical stirring in a water bath at 37 °C. Aliquots of AAE with a final concentration of 10 μg/mL dissolved in methanol were transferred to glass tubes and evaporated to dryness, except for the control samples without the inhibitor (AAE). After evaporation of the solvent, an incubation mixture with a volume of 100 μL was prepared, consisting of 5 pmol CYP2B6 or CYP3A4 enzyme, 50 mM potassium phosphate buffer (pH 7.4), and ultrapure water. An NADPH-generating system composed of 0.1 M G6P:10 mg/mL NADP^+^:1000 IU/mL G6PDH = 50:25:1 (*v/v/v*) was prepared immediately before use. This generating system contains glucose-6-phosphate dehydrogenase, which regenerates NADP^+^ to NADPH, keeping the concentration of cytochrome P450 co-enzyme (NADPH) constant during incubation. The reaction was started by adding the generating system (15% of the volume in the last incubation, *v/v*). In order to determine the enzyme residual activity, *S*-mephenytoin and testosterone (200 μM final concentration) were used. Then, 1 mL of ice-cold 1% solution of formic acid in dichloromethane was used to terminate the reaction. The samples were mixed and then centrifuged at 1900× *g* for 10 min. After centrifugation, two layers (water and organic layer) were formed and 850 µL of the organic layer was transferred to cuvettes and evaporated.

Next, 30 μL of methanol was used to dissolve the sample, which was analyzed using HPLC Agilent Zorbax SB C18 column (4.6 × 250 mm, 3 μm) from Agilent Technologies (Santa Clara, CA, USA). To test the activity of CYP2B6, a marker reaction of *N*-demethylation of *S*-mephenytoin to *S*-nirvanol was observed. The mobile phase consisted of acetonitrile and water in a ratio of 40:60, *v/v.* The analysis was performed isocratically. The flow was set at 1.0 mL min^−1^. The injection volume was adjusted to 10 µL. Chromatograms were recorded at 210 nm. The duration of the analysis was set to 20 min. The retention time of *S*-mephenytoin was 12.3 min and that of *S*-nirvanol was 9.3 min [25]. In the case of CYP3A4, hydroxylation of testosterone to 6β-hydroxytestosterone was observed. The mobile phase consisted of methanol and water in a ratio of 64:36, *v/v*. The analysis was performed isocratically. The flow was set to 1.0 mL min^−1^. The injection volume was set at 10 µL. Chromatograms were recorded at 254 nm. The duration of the analysis was set at 35 min. The retention time of testosterone was 19.2 min and that of 6β-hydroxytestosterone was 5.8 min [23]. In both cases, the amount of product obtained was measured as the area under the curve (AUC) relative to the control sample (without inhibitor). Clopidogrel and troleandomycin were used as positive controls for the CYP2B6 and CYP3A4, respectively. Clopidogrel reduced the CYP2B6 activity to 41.1 ± 0.8%, and troleandomycin reduced the CYP3A4 activity to 39.2 ± 1.1%.

### 2.4. Hemochromopyridine Assay

The hemochromopyridine experiment was used to determine whether or not heme might be destroyed by reactive intermediates produced by the cytochrome P450 cycle. It was carried out with some changes to the procedure published by Flink and Watson [26] and Paul et al. [27]. Hemin dissolved in DMSO (0.6 to 0.1 μM) was used to create a calibration curve. A wavelength between 500 and 600 nm was used to record the spectra. Then, 200 μL of the incubation mixtures containing 25 μM inhibitor (AAE) was prepared. The NADPH-generating system was added to the reaction to start it, and the incubation period was 30 min. After 30 min, the reaction was stopped by adding sodium hydroxide (final concentration 0.83 M) and pyridine (final concentration 0.06 M). Because the pyridine hemochromogen is unstable under basic circumstances, samples were taken and recorded on a spectrophotometer (UV-1280, Shimadzu Corporation, Kyoto, Japan) within 1 min of the addition of an alkaline solution [23]. Using the created calibration curve, the heme concentration in the samples was calculated. This analysis was carried out in triplicate. To avoid hydrogen peroxide being produced through an ineffective catalytic cycle, the incubations were repeated with CAT and SOD (5 IU each) added to the incubation mixture.

### 2.5. Reversible and Pseudo-Irreversible Inhibition Assay

One of the characteristics of the catalytic cycle of cytochrome P450 enzymes is the formation of covalent complexes with the ferrous form of iron. Pseudo-irreversible inhibitors have the possibility of rehabilitating enzyme activity after oxidation of heme iron with the use of oxidants. Therefore, three types of tests were conducted to test the pseudo-irreversible nature of the inhibitor: incubation mixture without AAE (control), incubation mixture with AAE, and incubation mixture with AAE to which an oxidant was added after incubation. Incubations lasted 30 min according to the conditions described earlier. After incubation, samples were dialyzed, and 20 mM potassium hexacyanoferrate was added to certain samples [28]. The cassettes were immersed in a 50 mM potassium phosphate buffer solution (pH 7.4) for 30 min (the dialysis solution was replaced three times). After dialysis, samples were transferred from the cassette back to the glass tube, and the residual enzyme activity was determined using *S*-mephenytoin and testosterone as marker substrates (200 μM final concentration). Since the NADPH-generating system was also dialyzed, it was again added to the incubations to initiate the enzyme reaction. Samples were incubated for 30 min with the same settings. An ice-cold solution of formic acid in dichloromethane was used to terminate the reaction process. HPLC was used to analyze the samples. In the case of CYP3A4, diltiazem was used to test out the pseudo-irreversible inhibition. A full recovery of enzyme activity was observed, and the results were used as positive control.

### 2.6. Statistical Analysis

All incubations in the assays in this study were done in triplicate. Residual activity calculations and statistical analysis were made using the program *R* (The R Project for Statistical Computing, Vienna, Austria) and Microsoft Excel (Microsoft, Redmond, WA, USA). Statistical significance between the samples and controls were tested using the Student’s *t*-test for the estimation of statistical difference (*p* < 0.05) and Mann–Whitney U-test was used to test the data normality.

## 3. Results

### 3.1. Enzyme Inhibition

**Figure 2:**
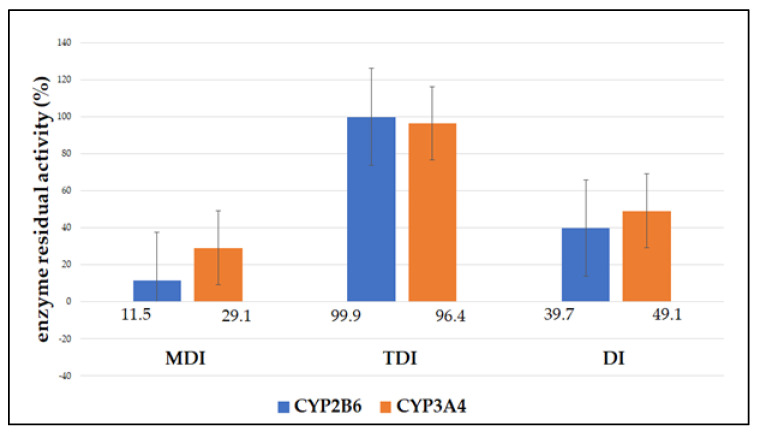
Residual activity (%) of CYP2B6 and CYP3A4 enzymes after incubation with AAE (10 μg/mL) expressed as mean of triplicate; MDI−metabolism-dependent inhibition; TDI−time-dependent inhibition; DI−direct inhibition.

Of all the observed types of inhibition, AAE most strongly inhibited the activity of CYP2B6 and CYP3A4 enzymes in the metabolism-dependent inhibition assay (Figure 2). In this experiment, the lowest activity of both enzymes was observed. CYP2B6 enzyme activity decreased by 88.5% and CYP3A4 enzyme activity decreased by 70.9% (Table 2).

The remaining activity of CYP2B6 was also significantly reduced in direct inhibition assays (49.1% of the remaining enzyme activity), whereas no significant inhibition was observed in the time-dependent inhibition assay (the remaining enzyme activity was 96.4%). The remaining activity of the CYP3A4 enzyme in the direct inhibition assay was also significantly reduced and amounted to 39.7%. No decrease in enzyme activity was observed in the study of time-dependent inhibition of CYP3A4. The remaining activity was 99.9%. Direct inhibition of the enzyme was also pronounced in the direct inhibition assay. Although the remaining activity of CYP2B6 and CYP3A4 enzymes was higher by 28.5% and 20.0%, respectively, the inhibition was still statistically significant (*p* < 0.05). The time-dependent inhibition assay showed no statistically significant inhibition of the enzymes (*p* > 0.05).

### 3.2. Heme Destruction

**Figure 3:**
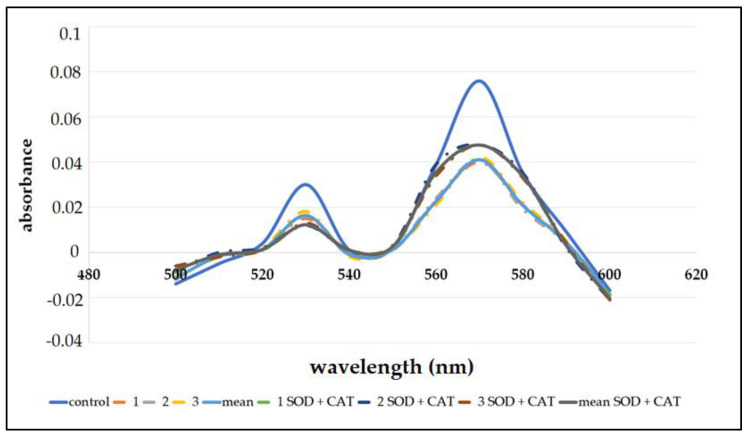
Spectra showing heme destruction with incubations containing AAE and AAE with SOD and CAT.

In order to test the potential binding of reactive metabolites to heme, a hemochrome pyridine assay was performed. It is suggested that the decrease in heme concentration is due to covalent binding of reactive metabolites to heme. In the cycles of cytochrome P450, reactive oxygen species can also be formed, which can also decrease heme concentration. However, to determine the destruction of heme concentrations by reactive metabolites of AAE, an assay was also performed using SOD and CAT. A parallel direction of the hemin solution was prepared, and heme concentrations of 0.63 μM and 0.59 μM were determined. Under reduced basic conditions, ferrous forms a complex with pyridine. Absorption maxima were observed at 531 nm and 570 nm. Incubation with AAE significantly reduced the heme concentration (Figure 3, Table 3).

The tests were confirmed by the addition of SOD and CAT. A statistically significant decrease in heme concentration was observed in both cases (*p* < 0.05). After the addition of SOD and CAT, the heme concentration was 53.2% and 61.8%, respectively, compared to the control (no AAE). These results confirm that the destruction in heme concentration is due to reactive metabolites in AAE.

### 3.3. Reversable and Pseudo-Irreversible Inhibition

**Figure 4:**
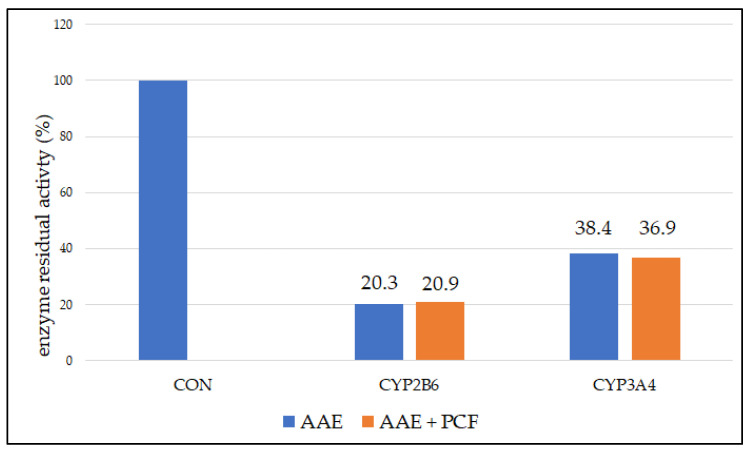
Residual activity (%) of CYP2B6 and CYP3A4 enzymes after incubation with AAE and dialysis and AAE with PCF and dialysis expressed as mean of triplicate; No statistically significant difference was observed between the samples with and without PCF (*p* > 0.05); CON—control.

In order to examine reversible and pseudo-irreversible inhibition, tests were performed with dialysis and the addition of an oxidant—PCF. In both cases, a statistically significant decrease in enzyme activity was observed (*p* < 0.05) (Figure 4).

The remaining activity of the CYP2B6 enzyme after incubation with AAE and dialysis was 20.3%, and the remaining activity of CYP3A4 was 20.9%. After dialysis and addition of PCF, the remaining activity of CYP2B6 and CYP3A4 was 38.4% and 36.9%, respectively. Reversible inhibition is characterized by the return of activity after dialysis. Pseudo-irreversible inhibition is characterized by the return of activity after dialysis and addition of an oxidant. In this case, no statistically significant difference was found between the two observed groups (*p* > 0.05). No reversible or pseudo-irreversible inhibition was observed.

## 4. Discussion

There are already numerous studies highlighting the importance of interactions of substances of natural origin with cytochrome P450 enzymes [29,30,31]. Although the therapeutic effects of *Artemisia annua* L. have been studied for many years, and this plant is used for nutritional and therapeutic purposes, its effect on CYP enzyme activity has been poorly studied. The authors de Magalhães et al. [32] investigated the effect of AAE in the form of tea infusions. The researchers noted the inhibitory effect of AAE on CYP3A4 enzyme activity with ketoconazole as a control, which is a known to cause complete inhibition of CYP3A4. In this study, inhibition of the CYP3A4 enzyme was observed, with the remaining enzyme activity ranging from 37% to 55%, depending on the type of AAE. Although the type of incubation tested was not described in detail, our results are somewhat consistent with the conclusions of the aforementioned study. In our study, an inhibition effect of 29.1% or 49.1% was achieved, depending on the type of inhibition. It is also important what type of extract is being tested, as this will determine the composition of the bioactive components in that extract. In our case, it was methanol extract. At this point, it should be emphasized that *Artemisia annua* L. is rich in substances of natural origin that can inhibit the activity of the CYP enzymes [13]. These are mainly sesquiterpenes [33], phenolic components [34], and coumarins [35]. Sesquiterpenes, such as artemisinin and arteannuin B; phenolic components, such as luteolin, quercetin, rutin, and apigenin; and coumarins, such as scopoline and scopoletin, are very poorly or almost not soluble in water and are soluble in organic solvents [36]. Therefore, the results of this study should also be considered in this context.

In this study, a decrease in CYP2B6 enzyme activity was observed by incubation with AAE. Enzyme activity decreased by 88.5% and 60.3%, respectively. These results are consistent with the only available research on the effect of AAE on the CYP2B6 enzyme conducted by Desrosiers et al. [37]. The effect of methanolic leaf extract of *Artemisia annua* L. on the activity of CYP2B6 and CYP3A4 enzymes in vitro was tested in comparison with artemisinin. The tested plant extract inhibited both CYP2B6 enzyme (IC_50_ = 6.07 µM) and CYP3A4 (IC_50_ = 4.93 µM) enzyme activity. In addition, this study compared the effect of *Artemisia annua* leaves in the form of tea on the activity of the above enzymes. The inhibition of the enzymes was also observed, namely IC_50_ = 2.31 µM for CYP2B6 and IC_50_ = 5.67 µM for the CYP3A4. In the case of the methanolic extract, the observed inhibitory effect was significantly stronger than that of artemisinin. This was also confirmed for the extract in tea form, except that no statistically significant difference in the inhibition CYP3A4 was observed in comparison with artemisinin at the highest tested dose of 600 µM. Although these studies were performed on human liver microsomes (HLM) and not on single isolated enzymes as in this case, positive agreement with the results of this study can be confirmed. In addition, further research should be directed toward the study of inhibition kinetics and the determination of kinetic parameters for individually studied enzymes, as well as the effect of AAE in in vivo studies.

The aforementioned could be explained by the recent results presented by Fu et al. [38]. They investigated the effects of orally administrated AAE in rats. After the ingestion, the authors detected scopolin, scopoletin, rutin, chrysosplenol D, casticin, arteannuin B, dihydroartemisinic acid, and artemisinic acid in rat plasma. Moreover, the authors investigated the binding affinity of some of these compounds and stated that coumarins and flavonoids have better binding affinity to CYP enzymes with a docking score > 5. This occurs mainly through forming hydrogen bonds and π–π stacking. The sesquiterpenes had weak interactions with proteins.

The most frequently observed type of inhibition of CYP enzymes is direct inhibition [39]. However, the most significant form of inhibition is metabolism-dependent inhibition, because it is the result of the formation of reactive metabolites that irreversibly bind to the enzyme, which leads to its inhibition. In this way, heme adducts can be formed. Therefore, in this study, the binding of AAE to heme was investigated to observe the potential destruction of heme. Destruction of heme concentration may also occur due to exposure to reactive oxygen species. Therefore, SOD and CAT are used to remove the influence of hydrogen peroxide and superoxide. We believe that the reactive forms of AAE bind irreversibly to heme in the 7th step of the catalytic cycle of the CYP enzyme [39]. In this study, a statistically significant decrease in heme was observed after the addition of SOD and CAT, being 53.2% and 61.8%, respectively. In comparison, benzbromarone, a known CYP3A4 inhibitor, causes 44% heme destruction by covalent binding to the prosthetic group and apoprotein [38]. The drug benzbromarone has been used as a powerful uricosuric. It is a non-competitive inhibitor of xanthine oxidase, and it was used to treat gout. The company that manufactured it has withdrawn this drug from the market due to its pronounced hepatotoxicity, but the drug is still approved as a generic in some countries around the world [40,41]. Ritonavir, another known CYP3A4 inhibitor, binds to heme iron via the thiazole nitrogen, forming a tight complex [42]. Therefore, the summary of product characteristics of ritonavir includes warnings of CYP3A4 inhibition and possible significant clinical effects when this drug is used with other drugs or xenobiotics administered concomitantly with ritonavir. Thus, irreversible binding of metabolites to heme may be clinically significant. In this regard, our study demonstrated the destruction of heme by incubation with AAE, an interaction not previously reported in the available literature. All health care professionals should be aware of the potential hazards of concomitant use of AAE with CYP3A4 or CYP2B6 substrates, and further research should be directed toward the development of in vivo interaction studies.

These results are particularly important in the context of the last test performed in this study, which confirms the irreversible nature of this inhibition. Enzyme dialysis after incubation failed to restore the activity of CYP2B6 and CYP3A4 enzymes. Addition of the oxidant PCF also did not result in a return of enzyme activity, confirming the view that this case is not a reversible or pseudo-irreversible inhibition. Irreversible inhibitors are much more dangerous in clinically relevant interactions because irreversible damage to the active site occurs in the form of covalent bonds. Such inhibition cannot be stopped by the addition of excess substrate. Therefore, the results of this study regarding the irreversibility of CYP2B6 and CYP3A4 inhibition have strong implications for further in vivo research.

## 5. Conclusions

AAE causes inhibition of the CYP2B6 and CYP3A4 enzymes in vitro. Inhibition was most pronounced in metabolism-dependent inhibition assays, followed by direct inhibition assays. No statistically significant enzyme inhibition was observed in time-dependent inhibition assays. In heme-binding assays, AAE was observed to cause a significant decrease in heme concentration, excluding the effect of reactive oxygen species on this decrease. The heme destruction is the result of binding of reactive AAE metabolites to heme, i.e., irreversible inhibition. Irreversible inhibition was confirmed by additional assays, whereas reversible or pseudo-irreversible inhibition could not be confirmed. Since *Artemisia annua* L. is widely used in traditional medicine, all health care professionals and stakeholders should be aware of this inhibition and the potential clinical implications. Further in vivo studies are needed to investigate the interactions of AAE with CYP2B6 and CYP3A4 enzyme substrates.

## Figures and Tables

**Table 1:** Main compounds found in *Artemisia annua* L.

Monoterpenes	Sesquiterpenes	Phenolic Compounds	Coumarins
1,8-cineole	artemisinin	quinic acid	scopolin
α-and-β-pinene	arteannuin B	caffeic acid	scopoletin
camphene	artemisinic acid	luteolin	
borneol		quercetin	
camphor		rutin	
carvone		apigenin	
limonene		isorhamnetin	
α-terpinene		kaempferol	
myrtenol		mearnsetin	
		artemetin	
		eupatorine	

Adjusted according to [13].

**Table 2:** Enzyme residual activity (%) after incubation with AAE in each individual test and incubation type.

**CYP2B6**			
**Incubation**	**MDI**	**TDI**	**DI**
1	11.1	99.8	39.9
2	12.1	101.2	40.4
3	11.5	98.9	38.9
Mean	11.5 ± 0.5	99.9 ± 1.1	39.7 ± 0.7
statistical significance	*p* < 0.05	*p* > 0.05	*p* < 0.05
**CYP3A4**			
1	29.9	95.4	48.8
2	29.1	96.7	48.2
3	28.2	97.1	50.2
Mean	29.1 ± 0.8	96.4 ± 0.8	49.1 ± 0.9
statistical significance	*p* < 0.05	*p* > 0.05	*p* < 0.05

**Table 3:** Heme concentration (%) after incubation with AAE and with AAE containing SOD and CAT.

Incubation	Heme Concentration (%)	
	Without SOD and CAT	With SOD and CAT
1	52.9	62.5
2	53.2	61.9
3	53.5	60.9
Mean	53.2 ± 0.3	61.8 ± 0.3
statistical significance	*p* < 0.05	*p* < 0.05

## Data Availability

The data that support the findings of this study are available from the first and corresponding author, M.K., upon request.
